# Health-Related Quality of Life (HRQOL) of Gastrointestinal Cancer Caregivers: The Impact of Caregiving

**DOI:** 10.31557/APJCP.2019.20.4.1191

**Published:** 2019

**Authors:** Nik Nairan Abdullah, Idayu Badilla Idris, Khadijah Shamsuddin, Nik Muhd Aslan Abdullah

**Affiliations:** 1 *Department of Community Health, Faculty of Medicine, Universiti Kebangsaan Malaysia, Jalan Yaacob Latif, Bandar Tun Razak, Cheras,*; 2 *Department of Public Health Medicine, Faculty of Medicine, University Teknologi MARA, Sungai Buloh,*; 3 *Oncology Clinic, Sunway Medical Centre, Sunway, Selangor, Malaysia.*

**Keywords:** Cancer, quality of life, caregivers, gastrointestinal, Malaysia

## Abstract

**Objective:**

This study examined the quality of life (QOL) of caregivers for gastrointestinal (GI) cancer patients, and associated factors.

**Methods:**

A cross-sectional study was conducted at three referral hospitals in Klang Valley, Malaysia. A total of 323 pairs of patients and caregivers from the oncology units of these hospitals completed questionnaires in Malay. The QOL of caregivers was measured using The Malay Caregiver Quality of Life questionnaire. The independent variables were caregiver and patient factors, care-related factors, the Caregiver Strain Index-Malay, and the Multidimensional Scale of Perceived Social Support-Malay. Simple and multiple linear regression analyses were performed to determine the factors associated with the QOL. Variables with p < 0.05 were considered significant in the multiple analyses.

**Results:**

Female caregivers were 68.1% of the total, and 46.4% caregivers were spouses to cancer patients. Their mean age was 44.50 (13.29) years old. About 51.7% were of Malay ethnicity. The mean score for QOL was 80.17 (21.58). Being a male caregiver (beta = 5.165, p = 0.011) and of Indian ethnicity (beta = -9.163, p = 0.001) were strongly associated with caregiver QOL. Male patients contributed higher QOL scores for the caregivers compared to female patients. There was an inverse relationship among caregiving strain, duration of caregiving, and caregiver QOL.

**Conclusion:**

The identification of factors that affect QOL will allow healthcare providers to develop appropriate interventions. It is important that caregivers be in good health so as not to compromise the care they provide to their patients.

## Introduction

Gastrointestinal (GI) cancer includes cancer of the esophagus, stomach, colon and rectum, liver, gallbladder, and pancreas. Colorectal cancer is one of the most commonly diagnosed cancers in men and women in Malaysia, being the number-one cancer in males and the second most common in females, after breast cancer (Azizah et al., 2015). 

Due to the increasing prevalence of GI cancer and advances in treatment, increasing numbers of patients with cancer are being treated as outpatients. Thus, the role of caregivers is becoming more significant because they contribute to informal care of patients in their homes. They have a role in giving drugs, managing side effects, reporting problems, keeping other family members informed of developments, and helping the patient decide on the best treatment (American Cancer Society, 2017). 

Cancer is a chronic illness that evolves throughout the patient’s lifetime. The trajectory of an illness will also affect the health-related quality of life (HRQOL) of the caregiver. The World Health Organization (2017) defined QOL “as an individual’s perception of their position in life in the context of the culture and value systems in which they live and in relation to their goals, expectations, standards and concerns.” It is a multidimensional concept, which incorporates physical and psychological health, as well as social well-being. 

The family caregivers of cancer patients experience more significant impairments than non-family caregivers (Aydogan et al., 2016). Caregiving for GI patients poses a larger challenge because of poor diagnosis, delay in detection, and the need for a high level of patient care (Shaw et al., 2013). Furthermore, caregivers of patients with upper GI cancer are at higher risk for psychological distress than those caring for cancers with longer disease trajectories, due to the poor prognosis of upper GI cancer (Shaw et al., 2013). Most family caregivers of patients with cancer are unprepared to support the burden of caregiving (Palma et al., 2012).

There is a conceptual framework for cancer caregivers (Fletcher et al., 2012) that identifies the factors that contribute to their QOL. These factors include sociodemographic factors (of both the caregiver and the patient), the caregiver’s health, and care-related factors. In addition, HRQOL tends to differ by sex (Son et al., 2012; Lim et al., 2016) and also age (Turkoglu and Kilic, 2012; Lim et al., 2016). A caregiver’s own health status also affects their QOL (Lu et al., 2010). Finally, the sex of the patient and the duration of cancer are also predictors of caregiver HRQOL (Li et al., 2016). 

Informal caregivers of lung cancer patients have been studied but less research has been performed on caregivers for GI cancer patients (Tan et al., 2018). In addition, few studies on the QOL of cancer caregivers have been performed in Malaysia, except for a few on caregivers for breast cancer (Nik Jaafar et al., 2014), hospice cancer, HIV/AIDS (Lua et al., 2013), and epilepsy patients (Lua et al., 2014). One such study reported that the QOL of caregivers to cancer patients in hospice care was lower than that of most Malaysians (Che Bakar et al., 2008). 

Furthermore, outside of Malaysia, the majority of research has been conducted on Caucasian populations. The findings of those studies, therefore, may not be directly applicable to the Malaysian population, which has a different socio-cultural and ethnic background. In addition, most published studies have examined caregivers as a broad category, not in relation to any cancer. Little is known about the QOL among Malaysian caregivers for patients with GI cancer knowing that some types of GI cancers have a high mortality rate. In addition to that, due to the lack of local data, we hypothesised that the QOL of caregivers is lower compared to the developed countries. Therefore, we would like to examine the HRQOL among the caregivers of GI cancer patients in combination with sociodemographic factors (of both caregivers and patients) and care-related factors. 

## Materials and Methods

*Study subjects*

This was a cross-sectional study, conducted from September 2017 to February 2018 at three major referral hospitals in Klang Valley, an area including the Federal Territory of Kuala Lumpur and surrounding districts. All Malaysian GI cancer patients aged 18 years old and older from the oncology units (including outpatient clinics, wards, and daycare) who had no reported history of mental illness were invited to participate. Each patient identified his or her caregiver. The caregivers were restricted to those who were 18 years old and older, and were requested to be those who had provided the most physical or emotional support during the illness, were free from cancer or any history of mental illness, and were able to understand English or Malay. The sample size (n) was calculated using the formula for two means which was n = . The parameters entered were = 1.96 for = 0.05, = 0.84 for 80% power, = standard deviation (SD) for male caregiver’s QOL which was 1.41, = SD for female caregivers’ QOL was 1.52. The estimated difference (d) was 0.5 (Lu et al., 2010). The required sample size was 269 pairs (patient and caregiver). However, with an addition of 20% non-respondents, the required sample size became 323 pairs. Purposive sampling was employed because we wish to focus on a particular characteristics of a population (GI cancer patients and their caregivers). This sampling was able to answer the study’s objectives. Two researchers approached the pairs, and those who were eligible were invited to participate. A total of 323 pairs gave written consent and completed self-administered questionnaires. Permission and approval to conduct the study was granted by the Medical Research and Ethics Committee, Ministry of Health Malaysia, NMRR ID:17-898-35896, and the ethics committee of the National University of Malaysia, UKM PPI/111/8/JEP-2017-433. 

*Instruments*

The caregivers were given a set of demographic questionnaires that requested the following data: age, sex, ethnicity, level of education, marital status, relationship to patient, employment, exercise status, smoking status, household income (in Malaysian ringgit = MYR), number of children less than 18 years old, number of chronic diseases, care-related factors (duration of caregiving [hours/week]), any shared caregiving, past caregiving experience), and responses to several scales (Caregiver Strain Index-Malay (CSI-M), Malay Multidimensional Scale of Perceived Social Support (MSPSS-M), and the Malay Caregiver Quality of Life scale (MCQOL).

From patients, information on age, sex, education level, employment, exercise status, smoking status, number of chronic diseases, cancer site (upper GI, lower GI, hepatobiliary), duration of cancer (months), cancer stages, and treatment received (surgery, radiotherapy, and chemotherapy) were collected.

**Table 1:** Study Population of Caregivers and Patients, N = 323 pairs

Independent Factors	Caregivers,n (%)	Patients,n (%)
Age (years)	Mean = 44.50 (13.29)	Mean = 59.59 (11.98)
	Range = 18–77	Range = 22–86
Sex		
Male	103 (31.9)	209 (64.7)
Female	220 (68.1)	114 (35.3)
Ethnicity		
Malay	167 (51.7)	-
Chinese	113 (35.0)	
Indian	35 (10.8)	
Other	8 (2.5)	
Education level		
No formal education	13 (4.0)	23 (7.1)
Primary	29 (9.0)	91 (28.2)
Secondary	139 (43.0)	152 (47.1)
Tertiary	142 (44.0)	57 (17.6)
Marital status		-
Unmarried	68 (21.1)	
Married	242 (74.9)	
Others	13 (4.0)	
Relationship to patients		-
Spouse	150 (46.4)	
Child	118 (36.6)	
Parent	30 (9.3)	
Others	25 (7.7)	
Employment		
Employed	195 (60.4)	70 (21.7)
Not employed	128 (39.6)	253 (78.3)
Exercise currently		
Yes	167 (51.7)	110 (34.1)
No	156 (48.3)	213 (65.9)
Smoking status		
Daily	28 (8.7)	12 (3.7)
Less than daily	13 (4.0)	13 (4.0)
Not at all	282 (87.3)	298 (92.3)
Household income (MYR)	Mean = 4216.97 (3659.122)	-
	Range = 350–16000	
Number of children < 18 years old	Mean = 0.83 (1.24)	-
	Range = 0–6	
Number of chronic diseases	Mean=0.54(0.88)	Mean=0.92(0.95)
	Range=0-6	Range=0-5
Primary cancer site		
Upper GI		65(20.1)
Lower GI	-	231(71.5)
Hepatobiliary		27(8.4)
Duration of cancer (months)	-	Mean=20.27(26.19)
		Range=17.40-23.14
Cancer stage	-	
1		16(4.9)
2		41(12.7)
3		93(28.8)
4		173(53.6)
Surgery	-	
Yes		264(81.7)
No		59(18.3)
Radiotherapy	-	
Yes		86(26.6)
No		237(73.4)
Chemotherapy	-	
Yes		215(66.6)
No		108(33.4)

**Table 2:** Characteristics of Care-Related Factors, CSI-M, and MSPSS-M

Factors	n (%)	Mean (standard deviation)	Range
Duration of caregiving (hours/week)	-	47.8 (33.27)	3.25–128.8
Shared caregiving			
Yes	239 (74)	-	-
No	84 (26)		
Had caregiving experience			
Yes	93 (28.8)	-	-
No	230(71.2)		
Caregiving Strain Index-Malay	-	4.62 (3.29)	0.00–13.00
Malay Multidimensional Scale of Perceived Social Support			
Significant others	-	5.74 (1.25)	1.00–7.00
Family		5.80 (1.13)	
Friend		4.80 (1.70)	

**Table 3:** The Mean Distributions of Domains in Malay Caregiver Quality Of Life (MCQOL)

Domains	Mean	SD	Range
Burden	22.47	8.43	0–40
Disruptiveness	20.55	6.39	0–28
Positive adaptation	20.21	5.51	1–28
Financial concerns	8.08	3.65	0–12
Others	21.25	5.89	2–32
Total	80.17	21.58	12.00–124.00

* Higher scores, better quality of life; *All items are reverse-scored except items 4, 10, 12, 16, 22, 27, 28, 34

**Table 4:** Factors associated with Caregiver Quality of Life (MCQOL)

	Simple Linear Regression			Multiple Linear Regression		
Independent variables	B	95% CI	p value	B	95% CI	p value
Indian caregivers vs. Malay	-17.443	-24.139,-10.746	<0.001	-9.163	-14.341, -3.985	0.001*
Caregiving Strain Index-Malay	-4.083	-4.646, -3.520	<0.001	-2.07	-2.929, -1.210	0.000*
Patient had surgery vs. no surgery	11.278	5.281, 17.275	<0.001	3.422	-1.278, 8.122	0.153
Caregiving duration	-0.003	-0.004, -0.002	<0.001	-0.001	-0.002, 0.000	0.025*
Male vs. female caregiver	7.851	2.848, 12.854	0.002	5.165	1.203, 9.127	0.011*
Age of cancer patient	0.323	0.129, 0.518	0.001	0.109	-0.039, 0.257	0.147
Male patient vs. female patient	7.523	2.641, 12.405	0.003	6.414	0.386, 12.442	0.037*
Caregiver exercise vs. no exercise	6.857	2.182, 11.532	0.004	2.037	-1.379, 5.454	0.242
Sharing caregiving vs. no sharing	9.815	4.720, 14.910	<0.001	1.405	-2.589, 5.399	0.489
Caregiver working vs. no working	-5.699	-0.902, -10.496	0.02	-0.111	-3.794, 3.572	0.953
Duration of cancer (months)	0.108	0.004, 0.002	<0.001	0.025	-0.042, 0.091	0.468
Caregiver other marital status vs. single	-15.633	-27.625, -3.641	0.011	-6.123	-14.757, 2.510	0.164
Low GI vs. upper GI	15.212	9.476, 20.949	<0.001	3.688	-0.412, 7789	0.078

Enter method: adjusted R^2^, 0.52; S.E, 15.03, *p value less than 0.05

The CSI-M is the Malay version of a self-rated, 13-item questionnaire that measures strain related to care provision (Othman and Teck, 2014). It consists of five major domains, covering employment, finances, social relationships, time, and physical well-being. The responses to all questions are limited to yes or no. Any yes response indicates that an intervention may be required. A total score of more than 7 indicates a high level of strain. The Cronbach’s alpha for this instrument was 0.80 in the present study.

The MSPSS-M is the Malay version of a questionnaire that assesses the perception of adequacy of social support from three sources: family, friends, and significant others (Ng et al., 2010). The MSPSS-M contains 12 items, making it simple to use and quick to administer and score (Zimet et al., 1988). The reliability of the questionnaire for our study was also good, with a Cronbach’s alpha of 0.91.

The QOL of the caregivers was measured using the MCQOL (Lua et al., 2013). This is an adaptation of the English version of the Caregiver Quality of Life scale (Weitzner et al., 1999). It consists of a total of 35 items. These items assess burden, disruptiveness, positive adaptation, financial concerns, and additional factors (disruption of sleep, satisfaction with sexual functioning, day-to-day focus, mental strain, being informed about the illness, protection of the patient, management of the patient’s pain, and family interest in caregiving). The reliability of the MCQOL for our study was good, with a Cronbach’s alpha of 0.90.

*Statistical analysis*

Statistical Package for the Social Science (SPSS) version 20.0 was used for data analyses. The descriptive analyses yielded frequency, percentages, means, and standard deviations for the characteristics of the patients and caregivers. The independent variables were sociodemographic characteristics and care-related factors, including patient characteristics and scores on the CSI-M and MSPSS-M. The dependent outcome was the total MCQOL score of the caregiver. For the domains of QOL, which were burden, disruptiveness, positive adaptation, financial concerns, and other, the central tendencies are presented as means, standard deviations, and ranges. General linear regression tests were used to identify variables that were independently associated with the caregiver’s QOL score in a simple linear regression. Variables with p < 0.05 were entered into multiple linear regression analyses. Variables with two-sided p < 0.05 were considered statistically significant. These significant variables are presented as unstandardized beta coefficients. 

## Results

*Study population characteristics*

Out of the 343 eligible pairs, 323 completed the questionnaires while 20 declined (response rate = 94.2%). Those who declined were not interested. The final study population of paired patients and caregivers is summarized in Table 1. A majority of caregivers were females, Malays, and employed. There were more spouses of patients among the caregivers than children or parents of patients. Most patients were diagnosed with lower GI cancer, followed by upper GI cancer. About 54% of these cancers were in stage 4. 

The mean duration of caregiving was 47.80 (33.27) hours per week, while the mean CSI-M score was 4.62 (3.29). The level of perceived social support was higher within the family domain than among significant others and friends (Table 2). Burden has the highest score of all domains in MCQOL (Table 3).

*Factors associated with Caregiver Quality of Life (MCQOL)*

Multiple linear regression analyses indicated that the sex and ethnicity of the caregiver, the duration of caregiving, and caregiver strain were significantly associated with caregiver QOL. For the patient’s factor, only sex of the patient was a significant predictor of caregivers’ QOL. Male patients gave caregivers’ QOL scores that were 7.52 points higher than female patients. Duration of caregiving contributed 10% (beta standardized = 0.096) to the QOL of the caregivers. Other patient characteristics were not significantly associated with the caregiver QOL (Table 4).

We conducted further analyses to check for multicollinearity and model fit, and there was no multicollinearity found. However, an interaction between the CSI-M and the sex of the patient was significantly associated with caregiver QOL. The variables in the final model explained 52% of the variation (adjusted R2 = 0.52, p < 0.001). The final model had a good fit, as indicated by the normal distribution curve. 

## Discussion

In the present study, the mean QOL score for caregivers was 80.17 (21.58). This score is lower than that found in Turkey (Turkoglu and Kilic, 2012; Wadhwa et al., 2013; Bilgin and Gozum, 2016; Lim et al., 2016). The diversity type of the cancer patients in previous studies can account for this discrepancy. Because our sample was limited to GI patients whose cancers were mostly in an advanced stage, the QOL of the caregivers could be expected to be lower. In Canada, patients were in better socio-economic status (Wadhwa et al., 2013) while in a Singaporean study, only outpatients were recruited (Lim et al., 2016). In addition, the hospital care provided to the Turkish stomach-cancer patients on chemotherapy referred to above may have indirectly contributed to the higher QOL in that study. However, the QOL of caregivers in a study of rectal cancer patients was lower than ours (Miguel et al., 2017). 

Nevertheless, overall, the QOL scores of the caregivers in our study were better than those in previous studies (Palma et al., 2012; Son et al., 2012; Effendy et al., 2015). The cancer patients in these earlier studies were all in an advanced stage, unlike our cancer patients, whose were in all stages. Compared to those previous studies, our study’s caregivers reported better scores for the burden domain of QOL. 

We found that male caregivers had higher QOL than female ones. This is consistent with studies in other countries (Awadalla et al., 2007; Wadhwa et al., 2013). QOL tends to differ by the sex of the caregiver (Kunzler et al., 2011). There are several reasons why a female caregiver might have an impaired QOL. First, women’s traditional roles include caregiving (Matthews, 2003). They may have the responsibility to care for other dependents, such as younger children and elderly parents. Beyond that, their financial resources tend to be scarce due to the loss of income from patient, which lowers their QOL. On the other hand, Lim et al., (2016) reported that male caregivers had an impaired QOL due to a poor balance between work and family. However, two studies (Son et al., 2012; Effendy et al., 2015) have found that the sex of the caregiver is not a significant predictor of QOL.

In our study, the caregivers of male cancer patients had better QOL than those caring for female patients. This is supported by a study conducted in Thailand which found that caregiver QOL was more strongly related to a patient’s characteristics than to the caregiver’s own characteristics (Warapornmongkholkul et al., 2018). There are also sex differences in coping with cancer (Hagedoorn et al., 2008). To support and care for others is often considered a core feature of the female sex identity (Hagedoorn et al., 2008). A study among female patients with stoma found to have worse QOL due to their psychological impairment (Krouse et al., 2009). In another study, higher stress levels were found in female GI cancer patients compared to male counterparts (Renemane et al., 2016). Female patients may feel distressed by needing to care for others even when they are unwell, due to their social role. This distress on the part of the patient may negatively impact the caregiver’s QOL. 

Furthermore, the significant interaction that we found between CSI-M and the sex of the patient supports the notion that patient’s sex impacts caregiver QOL. However, this finding is not in accordance with an Israeli study which reported that male colorectal cancer patients have a higher level of stress than female patients (Goldzweig et al., 2009). They were found to rely more on spousal support and less on others, and female caregivers had more external support from friends. However, Li et al., (2016) reported that the sex of the patient was not a significant predictor of caregiver QOL.

In the present study, the caregiver’s ethnicity was a significant predictor of their QOL. Asian caregivers’ QOLs have been found to be lower than those of other caregivers in European countries (Lim et al., 2016). Better QOL among caregivers in Western countries can be attributed to more accessible healthcare support systems. In many Asian and Muslim societies, caregiving is influenced by norms of filial piety (Gupta and Pillai, 2009). Although they assume an obligatory role towards their close family members, QOL may be affected by the structure of family dynamics and external support (Katbamna et al., 2004). 

In our study, Indian caregivers had lower QOL than Malays. We also found that Indians have lower mean incomes than Malays and Chinese. In addition, they also have lower rates of secondary and tertiary education. An earlier local study reported that QOL of Malay caregivers of breast cancer patients were significantly associated with caregivers’ mental health. This may be due to cultural meanings attached to caregiving and the cultural differences in support resources, faith, and religion for coping with care burdens (Haley et al., 2004). Caregiving experiences also depend on the systems of family dynamics and broader sociocultural and religious systems of belief (Baider and Goldzweig, 2012).

In the present study, the duration of caregiving contributed 10% to the QOL of the caregivers, and it was thus a significant predictor. This is similar to results found elsewhere (Wadhwa et al., 2013) although several other studies have found differently (Son et al., 2012; Li et al., 2016).

There was a significant inverse relationship between CSI-M and caregiver QOL. Greater strain was associated with lower QOL. This is in accordance with an Irish and a Turkish study (Donnelly et al., 2008; Turkoglu and Kilic, 2012). In the Irish study, when the level of strain was high among the caregivers of patients with esophageal cancer, mental health was worse. In addition, those caregivers reported greater levels of mental strain than the caregivers in our study (Donnelly et al., 2008). 

Finally, we found that the age of the caregiver was not a significant predictor of his or her QOL. This is similar to several studies outside of Malaysia (Son et al., 2012; Lim et al., 2016), although other studies (Turkoglu and Kilic, 2012; Effendy et al., 2015) have found otherwise. Employment (Zhu et al., 2013; Li et al., 2016), marital status (Turkoglu and Kilic, 2012), number of co-morbidities (Son et al., 2012), and duration of cancer (Li et al., 2016) have not been reported to be significant predictors for caregiver QOL; our findings are in line with those results.

This study had a few limitations. Our findings are only applicable to caregivers of patients who are receiving treatment at hospitals. However, there are cancer patients who were not undergoing active treatment at the hospitals. Their caregivers may have more issues of concern but we did not include them. The family structure of the culture was not studied. This could shed more light on how caregiving is appraised by those involved. We acknowledge that the physical conditions or symptom severities of patients, may influence the QOL of caregivers. The assessment of this may require more time from the participants as it uses a separate grading system and may discourage participation in the study. Because QOL is a subjective and dynamic measurement, it may change over the course of caregiving. Therefore, we suggest that a longitudinal study be conducted on this subject. 

Nevertheless, our study had many strengths. For example, major hospitals receive patients from most peninsular states in Malaysia. Our study was conducted locally and it documented that QOL related to a particular cancer type, which is a significant public health concern. Future research should explore this further, along with the coping skills of caregivers from different ethnicities.

Some of the caregivers reported lower QOL than others. Therefore, health care providers should be able to identify and offer more resources to the groups who are at risk,thus increasing their quality of life. The policymakers can provide a referral system for the caregivers for better health support, including psychological counselling and support groups. An emphasis on respite care can also help reduce strain on caregivers at risk for poor QOL. 

## Funding

There is no funding received for the study.

## Statement conflict of interest

There is no conflict of interest.
